# Chemical, microbial, and metabolic analysis of Taisui cultured in honey solution

**DOI:** 10.1002/fsn3.2185

**Published:** 2021-02-18

**Authors:** Yunjing Chen, Shuxiu Zheng, Guangwen Zhang, Jianming Luo, Junsheng Liu, Xichun Peng

**Affiliations:** ^1^ Department of Food Science and Engineering Jinan University Guangzhou 510632 China

**Keywords:** chemical components, metabolomics, microbial community, Taisui

## Abstract

Taisui, a special substance occasionally found in China, can now be artificially cultured. In order to evaluate the safety of an artificially cultured Taisui (acTS) and develop it into fermented, functional food or oral liquid, the macronutrients, trace elements, microbial community, and extracellular metabolites of Taisui have been investigated in this study. Results showed that the concentrations of total carbohydrates, protein, fat, total ash, and moisture of wet acTS were 2.13 g/100 g, 0.13 g/100 g, 0.07 g/100 g, 0.04 g/100 g, and 88.3%, respectively. The concentrations of top three trace elements of K, Ca, and P, are 1,424.92 mg/kg, 159.96 mg/kg, and 67.89 mg/kg, respectively. *Proteobacteria*, *Euryarchaeota,* and *Ascomycota* were the dominant phyla of bacteria, archaea, and fungi, respectively. *Uncultured_bacterium_f_Anaerolineaceae*, *Alcaligenes,* and *Ochrobactrum* were the three most abundant genera of bacteria; *Methanosaeta*, *Methanosphaera,* and *Natronomonas*, the most abundant genera of archaea; *Zygosaccharomyces*, *Mortierella,* and *Fusarium,* the most abundant genera of fungi. There were 311 metabolites increased in acTS. Most of the metabolites are beneficial to human. These metabolites can be contributed to microbes in acTS. In conclusion, acTS is not a good source of macronutrients and of trace elements, while the safeness of some microorganisms in acTS is also unknown. Nevertheless, it still provides some probiotics and beneficial metabolites for human. It is thus possible to develop acTS into foods when the safety of each microorganism is proved.

## Graphical abstract

Jelly‐like Taisui, a unique substance usually found in the wild in China, can be artificially cultured recently, and the chemical components, microbial structure, and metabolomics of the artificially cultured one were investigated to characterize its safety.

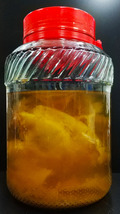

## 1 INTRODUCTION

Taisui, shaped and touched like meat or jelly, was named because of its morphological characteristics that are similar to the description of “Taisui” in ancient China. Firstly, Taisui being discovered in Shaanxi Province in 1992, China (Wang et al., 2017). Until 2013, Taisui had been reported 228 times. Among them, 98.57% of Taisui were accidentally discovered (Wang & Wang, 2014).

Taisui is usually considered as a complex of bacteria, fungi, and myxomycete (Sui, 2019). Yet, the dominant microbes in Taisui from various sources differ. For example, *Klebsiella oxytoca* and *Ralstonia eutropha* were both found by the plate‐culturing and nonculturing methods (Wang, 2007), whereas *Pseudomonas flulorescens* and *Brevundimonas mediterranea* were only detected from another Taisui sample (Tong et al., 2018). Of fungi, *Candida* and *Rhodotorula mucilaginosa* were identified by 18S rDNA sequencing method (Lin et al., 2013), whereas *Acremonium* and *Trichoderma* were the dominant fungi of another type of Taisui (Dai, 2007). In terms of myxomycete, *Didymium verrucosporum* and *Diderma deplanatum* have been successfully isolated using either corn meal‐agar or oat meal‐agar method (Dai, 2007). Moreover, archaea have also been found in Taisui and the dominant archaea were *Methanobacterium, Methanobrevibacter, Methanosphaera* (Wang & Wang, 2017). In addition, studies have investigated Taisui from the perspective of its chemical composition. Polyvinyl alcohol has been found as the main component of meat‐like Taisui, whereas polyacrylic acid or polyvinyl alcohol was the main component of jelly‐like Taisui (Li et al., 2020; Zheng & Dong, 2010). Some researchers believe that Taisui may have healthy benefits such as regulating immunity, inhibiting tumors, delaying aging, and eliminating fatigue. Within Taisui, PQQ (pyrroloquinoline quinone), nucleic acids, trace elements etc., have also been found as effective ingredients (Wang, 2018). In order to obtain the functional substances secreted from Taisui, Taisui has now been successfully artificially cultured, some of which are already on the market. For example, commercial Taisui is usually consumed with water or Chinese liquor, or the mixture of brown sugar and herbs (e.g., wolfberry, astragalus, and jujube). Taisui can also be used in food industry. For example, *Lactobacillus* and *Aspergillus*, isolated from Taisui (Dai, 2007; Han et al., 2018), can be potentially used in the brewing and soy sauce industry (Fang et al., 2006).

The chemical and microbial compositions of Taisui vary with the source of Taisui (Li et al., 2015; Wang, 2018). Consequently, the chemical compositions, total carbohydrates, protein, fat, total ash, moisture, and trace elements of Taisui cultured in honey solution have been investigated, as well as the microbial structure and extracellular metabolites of its bacteria, archaea, and fungi in this manuscript. Its aim is to fully understand the safeness of acTS products.

## 2 MATERIALS AND METHODS

### 2.1 Material

**FIGURE 1:**
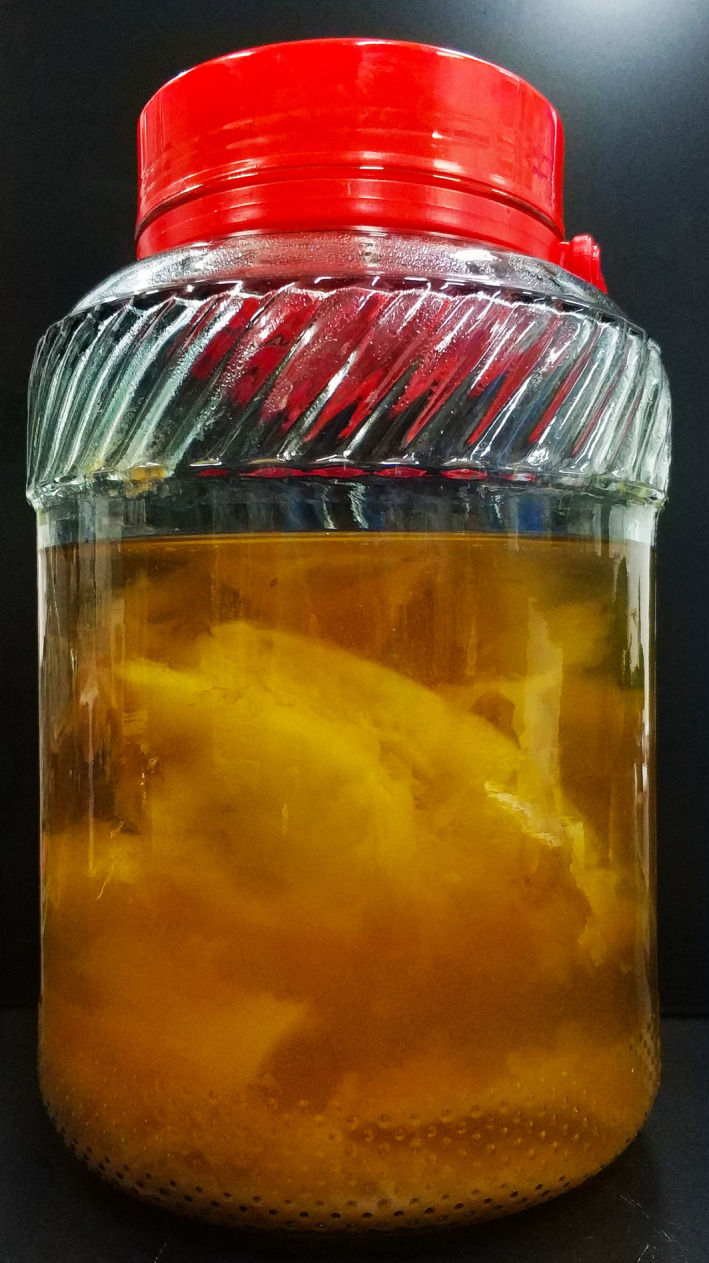
Artificially cultured jelly‐like Taisui

The mixture of Taisui and honey solution (Figure 1) was kindly gifted by Guangzhou KingCell Co., Ltd. Taisui sample is light yellow, cream, and transparent. It is cultured in honey solution at room temperature (about 25°C) with a ratio of honey to water of 6:1 (v/v).

### 2.2 Chemical components analysis

Wet acTS was washed by distilled water and then was homogenized in a pulverizer. All tests were performed in triplicate.

The concentrations of total carbohydrates, protein, fat, total ash, moisture, and trace elements were determined by phenol‐sulfuric acid method (Albalasmeh et al., 2013), Kjeldahl method (Oftedal et al., 2014), acid hydrolysis method (Lakshanasomya et al., 2011), burning gravimetric method (Oellig et al., 2020), oven drying method (Chiachung, 2003), and ICP‐AES (Inductively Coupled Plasma Atomic Emission Spectrometry) method (Bitter et al., 2020).

### 2.3 Microbial composition of bacteria, archaea and fungi

Due to the difference in appearance, we divided the artificially cultured Taisui solution into three parts: the upper, the lower, and the medium part (denoted as group U, group L, group M, respectively). Group U and group L were washed by 75% ethanol solutions and distilled water. All tests were performed in triplicate.

DNA from the three groups was extracted using MN NucleoSpin 96 Soil Kit (Macherey‐Nagel). Sequencing was analyzed on the Illumina Mi Seq platform (Illumina MiSeq). The sequences were clustered at a similarity level of 97% (USEARCH, version 10.0) (Edgar, 2013), and the OTUs were filtered with 0.005% of the number of all sequences as a threshold (Bokulich et al., 2013). Microorganisms with less than 0.1% of RA were classified into “Others.”

Phylogenetic Investigation of Communities by Reconstruction of Unobserved States (PICRUSt) software (version1.0.0^6^) was used to predict functional genes composition. The obtained OTU was standardized. According to the unique greengene id corresponding to each OTU, the KEGG family information and the abundance of KEGG were obtained. The abundance of each type of function was obtained from KEGG database.

### 2.4 Metabolomics analysis

Cultured Taisui honey solution was set as culture group (denoted as group Cul), and the uncultured Taisui honey solution was set as control group (denoted as group Con). All tests were performed in sextuplicate.

Samples were mixed (v/v, 1:5) with 80% methanol aqueous solution and were collected for analysis using a Thermo Scientific Vanquish UHPLC system with a Thermo Hyperil Gold (C18) column coupled with a Mass Spectrometer detector Q Exactive HF‐X (Thermo) (Dunn et al., 2011). Compared with mzCloud database, the data recognition and quantitative results were obtained. Metabolites were determined as compounds with the Variable Importance in Projection (VIP) value > 1 and *p* <.05 and Fold Change value (FC) >2 or <0.5 (Svenja et al., 2016).

### 2.5 Statistical analysis

Statistical analysis was performed with SPSS software (IBM, version 22.0). Results were presented as mean ± standard deviation. Two‐tailed T tests were conducted to compare the difference among different groups. Pearson correlation was performed with SPSS software (IBM, version 22.0), and heat maps were performed with Origin (OriginLab, version 2018). Statistical significance was set at a *p* value < .05.

## 3 RESULTS

### 3.1 Potential macronutrients and trace elements

As shown in Tables 1 and 2, the total content of carbohydrate was 2.13 g/100 g and was the main nutrient of wet acTS compared with other macronutrient. The protein and fat contents were 0.13 g/100 g and 0.07 g/100 g, respectively. Total ash was the lowest component of solid with a number of 0.04 g/100 g. The moisture concentration was the highest with a number of 88.3%. In total, 15 kinds of trace elements were detected. K was the most abundant (1,424.92 mg/kg), followed by Ca, P, Fe, Mg, and Al within 11–160 mg/kg. The concentrations of macronutrients and trace elements mentioned above were expressed as wet weight.

**TABLE 1:** Content of carbohydrate, protein, fat, total ash, and moisture of wet acTS

Carbohydrate g/100 g	Protein g/100 g	Fat g/100 g	Total ash g/100 g	Moisture %
2.13 ± 0.02	0.13 ± 0.00	0.07 ± 0.00	0.04 ± 0.00	88.30 ± 0.00

**TABLE 2:** Concentration of trace elements of wet acTS

Elements	*c* (mg/kg)	Elements	*c* (mg/kg)	Elements	*c* (mg/kg)
Al	11.72	Cu	0.24	Ni	0.31
B	0.26	Fe	42.92	P	67.89
Ba	0.43	K	1,424.92	Pb	1.52
Ca	159.96	Mg	27.08	Sr	0.67
Cr	0.89	Mn	1.38	Zn	2.02

### 3.2 Microbial composition of bacteria, archaea, and fungi

#### 3.2.1 OTU, alpha‐diversity indices and Venn diagrams

The number of OTU and the alpha diversity indices are shown in Table 3. The OTU numbers and alpha diversity indices indicated that no significant differences were found between group U, L, and M in terms of bacteria and archaea. For fungi, the OTU number, Shannon index, ACE index, and Chao1 index of group L were significantly higher than those of other two groups, and Simpson index was significantly lower than those of other two groups.

**TABLE 3:** Alpha diversity indices of different groups of bacteria, archaea, and fungi

Group	OTU	Shannon	Simpson	ACE	Chao 1
Bacteria					
U	1,630	6.04 ± 0.01	0.0082 ± 0.0005	1,306.64 ± 34.14	1,311.31 ± 31.40
L	1,569	6.12 ± 0.04	0.0073 ± 0.0000	1,355.90 ± 58.51	1,364.20 ± 52.96
M	1,550	6.03 ± 0.04	0.0086 ± 0.0000	1,318.27 ± 98.95	1,328.34 ± 93.62
Archaea					
U	32	2.05 ± 0.08	0.1511 ± 0.0106	21.46 ± 3.85	18.83 ± 3.63
L	28	2.11 ± 0.11	0.1557 ± 0.0155	26.38 ± 13.19	18.67 ± 1.20
M	29	1.87 ± 0.20	0.2182 ± 0.0413	14.34 ± 7.24	18.67 ± 0.33
Fungi					
U	417^a^	1.26 ± 0.41^a^	0.6266 ± 0.1105^a^	191.67 ± 49.28^a^	194.00 ± 51.56^a^
L	795^b^	4.73 ± 0.04^b^	0.0498 ± 0.0004^b^	647.22 ± 14.64^b^	647.21 ± 14.79^b^
M	475^a^	2.28 ± 0.16^a^	0.4462 ± 0.0349^ab^	228.17 ± 6.78^a^	232.17 ± 9.29^a^

The Venn diagram indicated the shared OTUs and unique OTUs of bacteria, archaea, and fungi respectively. In the Venn diagram, there are 1,411 shared OTUs in bacteria, 15 shared OTUs in archaea and 186 shared OTUs in fungi. The shared OTUs accounted for large part of OTUs (Figure S1).

#### 3.2.2 Composition of microbiota at the phylum level

**FIGURE 2:**
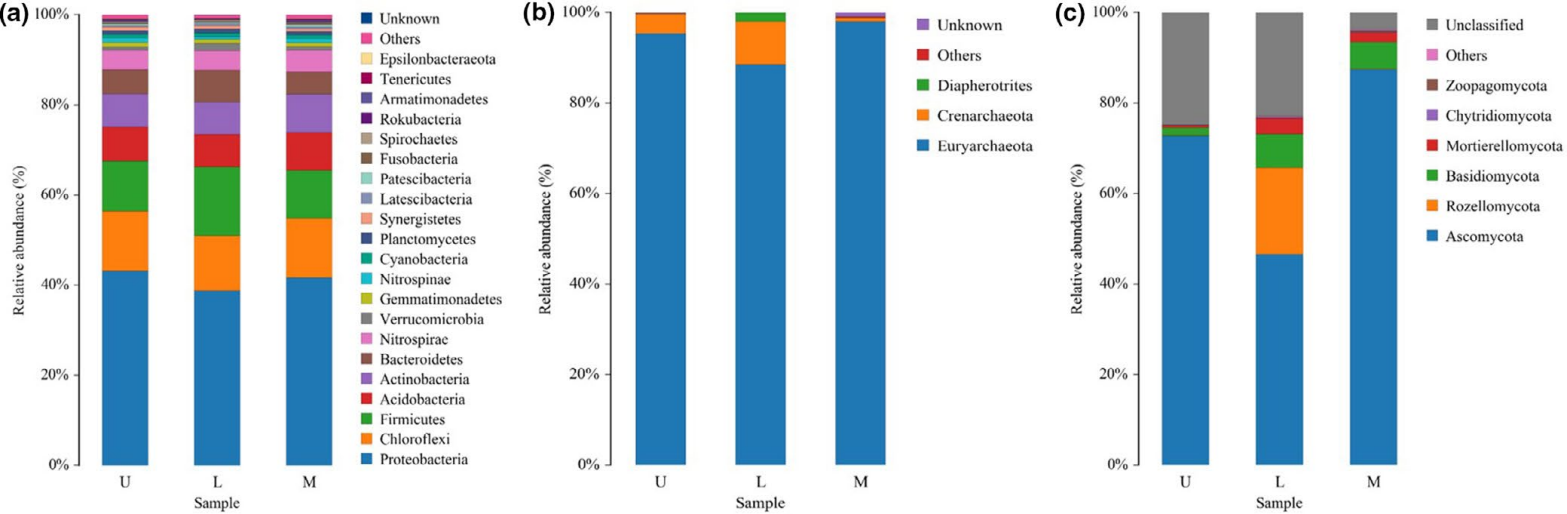
Composition of bacteria (a), archaea (b), and fungi (c) at the phylum level

The microbial community structure of bacteria, archaea, and fungi at the phylum level was presented in Figure 2, and the corresponding relative abundance (RA) was listed in Table S1.

The RA of *Proteobacteria* was the highest in L, U, and M groups, accounting for 43.10%, 38.73%, and 41.70%. Followed by *Chloroflexi*, *Firmicutes*, *Acidobacteria*, *Actinobacteria*, *Bacteroidetes,* and *Nitrospirae*, these phyla of bacteria were the micro part of bacteria and their RAs were 15%–4%.

The dominant phylum of archaea among 3 groups was *Euryarchaeota* (>88%), while *Crenarchaeota* and *Diapherotrites* only accounted for a low ratio of the community < 10%), respectively.

However, only *Ascomycota* was predominant among fungi with higher than 46% RA among 3 groups. The RA of *Rozellomycota* was 19.09% in group U. The RA of *Basidiomycota* and *Mortierellomycota* was 7.47% and 3.45% in group U, respectively, and was 6.05% and 2.09% in group M. The RA of *Basidiomycota* only accounted for 1.77% in group L.

#### 3.2.3 Composition of microbiota at the genus level

**FIGURE 3:**
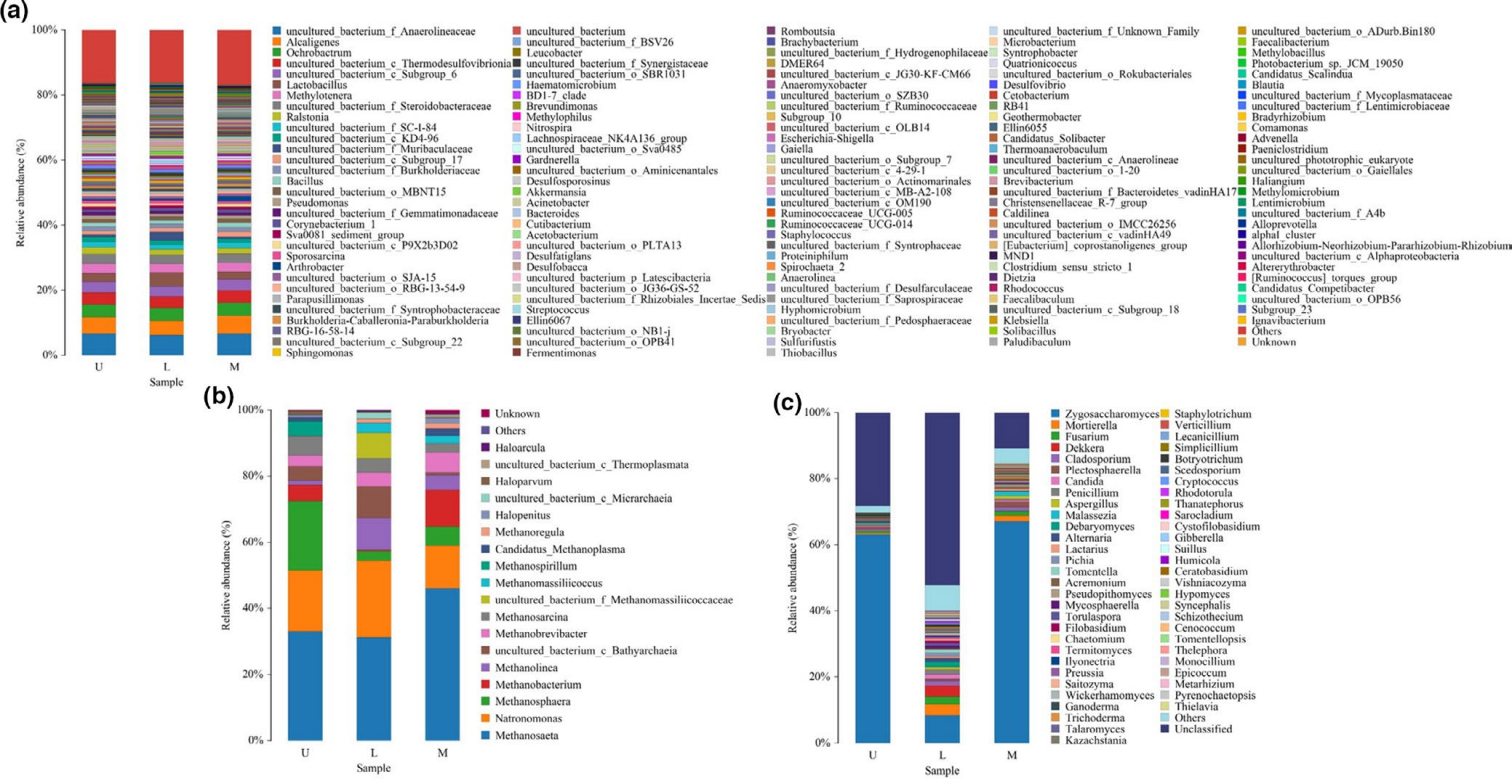
Composition of bacteria (a), archaea (b), and fungi (c) at the genus level

The microbial community structure of bacteria, archaea, and fungi at the genus level was presented in Figure 3 while the top 10 relative abundance (RA) was listed in Table S2.

The dominant bacterial genera of Taisui community were *uncultured_bacterium‐ _f_Anaerolineaceae*, *Alcaligenes*, *Ochrobactrum*, *uncultured_bacterium_c_Thermodsulfovi‐ brionia,* and *uncultured_bacterium_c_Subgroup_6*. The RA of all these genera was about 22%. The RA of *uncultured_bacterium_f_Steroidobacteraceae*, *Methylotenera*, *Lactobacillus*, *Ralstonia,* and *uncultured_bacterium_f_ SCI‐84* together was less than 14% in the three groups.

*Methanosaeta* and *Methanosphaera* were the predominant genera in group L with a RA of 32.03% and 20.63%, respectively. *Methanosaeta* and *Natronomonas* were the dominant genera in group U with a RA of 30.11% and 23.75%, respectively, and were also the dominant genera in group M, and their RAs were 35.35% and 20.40%. *Uncultured_bacterium_c_Bathyarchaeia*, *Methanobacterium*, *Methanobrevibacter*, *Methanosarcina*, *Methanolinea,* and *Methanomas‐siliicoccus* were also the major part of archaea community.

*Zygosaccharomyces* was the most abundant genus among three groups with RA of 63.17%, 8.23%, and 66.61%. Followed by the genus of fungi *Plectosphaerella*, *Fusarium*, *Cladosporium*, *Mortierella*, *Candida*, *Penicillium*, *Chaetomium*, and *Pseudopithomyces*. However, the RA of these genera of fungi only accounted for about 3.33%–0.20%.

#### 3.2.4 Functional genes prediction

**FIGURE 4:**
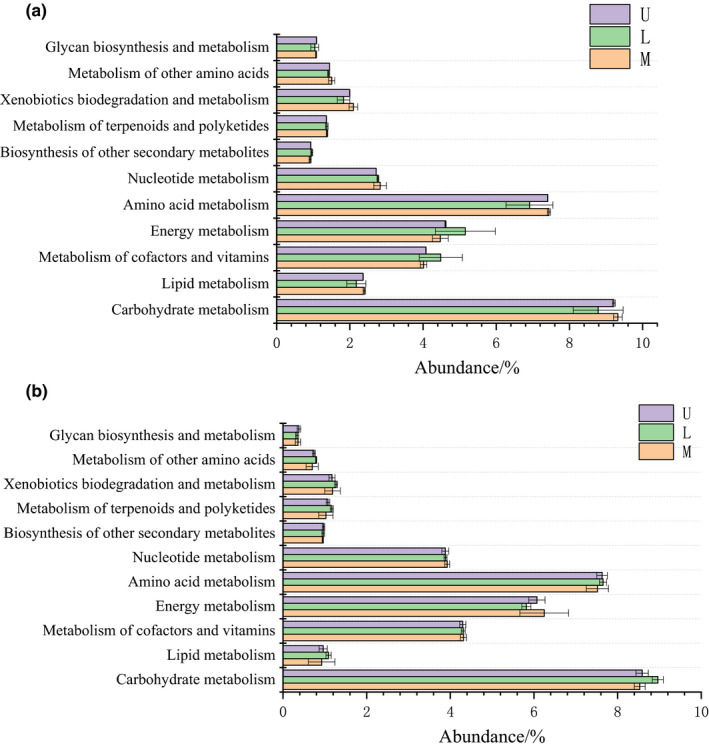
PICRUSt functional predictions of the bacteria (a) and archaea (b)

Based on KEGG database, PICRUSt analysis is used to predict functional genes composition by comparing species composition obtained from the sequencing data. As shown in Figure 4, it was found that the functional genes of Metabolism was the most dominant category. The KEGG level 2 result showed that group U, L, and M had high abundance of carbohydrate metabolism, amino acid metabolism, energy metabolism, metabolism of cofactors and vitamins, and nucleotide metabolism among bacteria and archaea. The abundance of those functional genes categories respectively accounted for more than 27% and 30% of the bacteria and archaea. However, group U, L, and M had similar abundance in the same category and showed no significant difference among three groups.

### 3.3 Extracellular metabolites

The metabolites in group Cul were analyzed. The KEGG pathway and related metabolites were presented in Figure S2 and Table 3. A total number of 720 metabolites were detected, among which 311 metabolites were upregulated and 178 metabolites were downregulated. The FC of some metabolites was showed in Table S3.

Most metabolites were related to environmental information processing, metabolism, and/or organismal systems, whereas only a small part were related to drug development and/or genetic information processing. Some of metabolites are functional components such as gallic acid and quercetin. The metabolites could be divided into the following nine categories: (a) Carbohydrate, such as sucrose and stachyose. (b) Amino acid, such as *L‐*phenylalanine, *L‐*glutamic acid, and *L‐*cystathionine. (c) Vitamin, such as vitamin A, pantothenic acid, and *L‐*ascorbate. (d) Nucleotides, such as adenine, cytosine, and thymine. (e) Alkaloid, such as theophylline, betaine, and capsaicin. (f) Amines, such as phenethylamine, tyramine, and 3‐methoxytyramine. (g) Acids, such as kinic acid, syringic acid, and ferulic acid. (h), Phenols, such as kaempferol, *o‐*cresol, and xanthohumol. (i) Aldehydes, such as cuminaldehyde, 3,4‐dihydroxybenzaldehyde, and phenylacetaldehyde.

### 3.4 Pearson correlation between the microbes and metabolites

**FIGURE 5:**
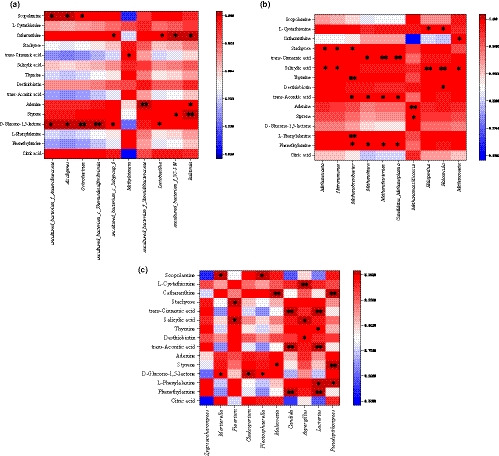
Pearson correlation between the metabolites and bacteria (a), archaea (b), and fungi (c)

Pearson correlation heat map showed the correlation between 10 microbes with the highest abundance and the 10 metabolites with highest abundance and five metabolites with lowest abundance (Figure 5).

*Methanobrevibacter*, *Methanolinea*, *Methanobacterium*, *Candidatus_Methanoplasma*, *Candida,* and *Lactarius* showed significant positive correlation with Phenethylamine and *trans*‐Aconitic acid*. Methanobrevibacter*, *Lactarius,* and *Pseudopithomyces* showed significant positive correlation with *L*‐Phenylalanine. *Alcaligenes*, *Ochrobactrum*, *Lactobacillus*, *Cladosporium,* and *Plectosphaerella* showed significant positive correlation with *D*‐Glucono‐1, 5‐lactone. *Ralstonia*, *Methanomassiliicoccus*, *Malassezia,* and *Pseudopithomyces* showed significant positive correlation with Styrene. *Ralstonia* and *Methanomassiliicoccus* showed significant positive correlation with Adenine. *Haloarcula* and *Aspergillus* showed significant positive correlation with Desthiobiotin. *Methanobrevibacter* and *Lactarius* showed significant positive correlation with Thymine. *Methanosaeta*, *Natronomonas*, *Halopenitus*, *Haloarcula*, *Methanosaeta*, *Fusarium,* and *Aspergillus* showed significant positive correlation with Salicylic acid. *Methylotenera*, *Methanolinea*, *Methanobacterium*, *Candidatus_Methanoplasma*, *Candida,* and *Lactarius* showed significant positive correlation with *trans*‐Cinnamic acid. *Methanosaeta*, *Natronomonas*, *Methanobrevibacter,* and *Fusarium* showed significant positive correlation with Stachyose. *Lactobacillus*, *Ralstonia*, *Methanosaeta*, *Malassezia,* and *Pseudopithomyces* showed significant positive correlation with Catharanthine. *Halopenitus*, *Haloarcula,* and *Aspergillus* showed significant positive correlation with *L*‐Cystathionine. *Alcaligenes*, *Ochrobactrum*, *Mortierella*, and *Plectosphaerella* showed significant positive correlation with Scopolamine. In conclusion, Pearson analysis indicated that the metabolites can be contributed to these microbes.

## 4 DISCUSSION

In this study, the carbohydrate, protein, fat, total ash, and moisture content were 2.13%, 0.13%, 0.07%, 0.04%, and 88.3%, respectively, in the wet acTS. *Proteobacteria*, *Euryarchaeota,* and *Ascomycota* were the dominant phyla of bacteria, archaea, and fungi, respectively. *Uncultured‐_bacterium_f_Anaerolineaceae*, *Alcaligenes,* and *Ochrobactrium* were the three most abundant genera of bacteria; *Methanosaeta, Methanosphaera,* and *Natronomonas* of archaea; *Zygosacch‐ aromyces*, *Mortierella,* and *Fusarium* of fungi. The contents of 311 metabolites increased, and the contents of 178 metabolites reduced in the acTS. Many of them were related to environmental information processing, metabolism, and organismal systems of KEGG pathway.

Our results showed that the acTS contains 2.13% carbohydrate, lower than that in ordinary food. The protein content of Taisui was significantly lower than the normal wild type of wet Taisui and also the standard of “high protein food” (12%) (Zhang Tao et al., 2018). In the meantime, the fat content was also significantly lower than that of the normal wet wild‐type Taisui and the standard of “low fat food” (3%) (Chen et al., 2010). The concentration of total ash and trace elements was far lower than the normal concentration range of wet wild‐type Taisui (Zhu et al., 2011). In short, the acTS used in current study is a substance with low carbohydrate, low protein, low fat, low total ash, low trace elements, and high moisture, while wild Taisui is a substance with high protein. The differences between these two types of Taisui may due to their culture environment. Since all of carbohydrate, protein, fat, total ash, and trace elements were low, which means acTS is not a good source of macronutrients and trace elements.

In order to investigate whether or not it is safe to consume acTS as food ingredient, we have thus investigated the composition of microorganism in the acTS. In terms of bacteria, *Proteobacteria* contains *Alcaligenes, Ochrobactrum, Methylotenera,* and *Ralstonia*, and *Firmicutes* contains *Lactobacillus*. These genus bacteria were dominant in this acTS as well. *Alcaligenes*, *Methylotenera*, *Ochrobactrum*, and *Ralstonia* usually are pathogenic microorganism (Kalyuzhnaya et al., 2010; Li, 2018; Qi et al., 2019). *Lactobacillus*, the dominant bacteria of wild‐type Taisui (Han et al., 2018), was also found. *Lactobacillus* is a well‐known probiotic parasitizing in intestine and vagina (Setiarto et al., 2017). This acTS contains potential pathogenic bacteria and probiotics. However, it cannot infer that there is a safety risk in this acTS, because the safeness is not consistent for different bacterial strains. (Arellano et al., 2020).

In terms of archaea, *Euryarchaeota* contains many species including *Methanosaeta*, *Methanosphaera*, *Natronomonas*, *Methanosarcina*, *Methanobacterium*, *Methanospirillum*, *Methanobrevibacter, and Methanolinea*, which were the dominant genera of archaea in acTS. Previous report found that *Methanobacterium*, *Methanobrevibacter,* and *Methanosphaera* were the major part of wild‐type Taisui (Wang & Wang, 2017). Abundance of methanogenic archaea in patients with gastrointestinal and metabolic diseases was larger than normal people, which may be potentially detrimental for host health (Guo et al., 2015). However, it is still difficult to evaluate the safeness of consuming food with these archaea as the relationship between archaea and human health is not studied well.

For fungi, most of the dominant genera belong to *Ascomycota*, such as *Zygosaccharomyces*, *Fusarium*, *Cladosporium*, *Candida*, *Penicillium*, *Chaetomium*, *Debaryomyces*, *Alternaria*, *Pichia*, *Aspergillus* and *Acremonium*. *Zygosaccharomyces*, *Dekkera*, *Debaryomyces,* and *Aspergillus glaucus* are widely used in fermentation (Deng et al., 2009; Fang et al., 2006; Ishchuk et al., 2016; Zhang et al., 2016; Zhu et al., 2003). Dai (2007) reported that *A. glaucus* was the dominant fungi in wild Taisui. *Lactarius*, belonging to Chordata, can produce chemicals such as sesquiterpenes and has the activity of anti‐tumor (Barros et al., 2007). Fortunately, these fungi are relatively safe because they are widely present in traditional foods. In addition, there are some commonly used industrial species in acTS, such as *Mortierella* for *n*‐6 polyunsaturated fatty acids production, *Pichia* for protein, *Chaetomium* for cellulase, and *Penicillium* for penicillin (Ahmad et al., 2014; García‐Estrada et al., 2020; Ho et al., 2007; Sun et al., 2019). The safeness of some genera of fungi in acTS is still undefined. *S*everal types of *Alternaria* could cause diseases, but it could produce anticancer drugs such as vinblastine (Duan et al., 2008). Some genera of fungi are relatively unsafe. For example, *Fusarium* could produce toxin (Geiser et al., 2004); some species of *Cladosporium* can cause allergy (Bensch et al., 2012); *Candida* could cause inflammation (Tarang et al., 2020) and *Malassezia* could cause dandruff (Sommer et al., 2015). So, this acTS contains potential pathogenic and probiotics. However, it also cannot infer that the acTS has a safety risk because different strains of fungi, even belonging to the same species, have different safety (Arellano et al., 2020).

Due to the existence of potential unsafe microorganism in acTS, acTS metabolites were further investigated. The metabolites of acTS involve a large part of KEGG pathways, except for Cellular Processes and Human Diseases. Different metabolites have different functions. According to the function of the increased metabolites, they can be divided into the following five categories: (a) Improving color, fragrance, and taste of food, such as *L‐*glutamic acid, vanillyl alcohol, and cuminaldehyde; (b) Preventing the damage of spoilage bacteria and pathogenic bacteria, such as protocatechuic acid, quercetin, and luteolin. Luteolin have the antibacterial ability, and the higher the concentration, the more the antibacterial; (c) Enhancing nutrition and maintaining healthy, such as ferulic acid, desthiobiotin, and deoxyadenosine. The herbs that full of ferulic acid is used for curing thrombosis in China for a long time (Ou & Kwok, 2004). Desthiobiotin could have the influence of gene expression like biotin (Rodriguez‐Melendez et al., 2003); (d) Anticancer, anti‐inflammatory, anti‐diseases, anti‐oxidation, such as kaempferol, chlorogenic acid, ferulic acid, genistein, luteolin, naringenin, gallic acid, protocatechuic acid, quercetin, *L‐*ascorbate, genistein, luteolin, and cytidine. Genistein could inhibit the carcinogenesis in animal models (Banerjee et al., 2008). Quercetin has excellent antioxidant capacity in vitro within the flavonoid family (Boots et al., 2008); (e) Chemical and pharmaceutical intermediates or product, such as salicylic acid, terephthalic acid, styrene, thymine, cytosine, hypoxanthine, uracil, 4‐hydroxybenzal‐dehyde, *o‐*cresol, 4‐hydroxyphenylacetic acid, and 3,4‐dihydroxybenzaldehyde. 4‐hydroxybenzaldehyde is artificially synthesized into drug with anti‐inflammatory activities (Lim et al., 2008). Salicylic acid is pharmaceutical intermediates, which is used for anti‐inflammatory medicine like aspirin (Su et al., 2017). In conclusion, most of the metabolites are beneficial to human.

The KEGG pathways of functional genes prediction and extracellular metabolites were consistent. Pearson correlation analysis showed that some microbes are significantly associated with the above metabolites. According to previous report, *Aspergillus* is one of the most important strains commercially produced citric acid by starch or sucrose‐based medium fermentation (Aboyeji et al., 2020). It is known that honey contains a high amount of sucrose and thus is a good source for *Aspergillus* to produce citric acid. As reported, *Candida* could also be potentially used to produce citric acid (Uzah et al., 2020). *Aspergillus* produces *D*‐biotin with a structure similar that of desthiobiotin, suggesting that *Aspergillus* may potentially to produce desthiobiotin (Zheng, 2007). *Candida* can utilize its own lipase to catalyze phenylethylamine synthesis from lipid substrate (Wen, 2012). Except for *Candida*, the main organisms commonly used to produce stereoselective lipases are *Pseudomonas*, *Fusarium,* and *Aspergillus* (Qin, 2006). Therefore, microbes may also be responsible for the production of metabolites. This study indicates that the acTS used in current study could be potentially developed into fermented food, functional food, or oral liquid.

## 5 CONCLUSION

In current study, the chemical compositions, the microbial structure of bacteria, archaea, and fungi, and the extracellular metabolites of acTS were analyzed. The concentrations of macronutrient and trace elements were low, indicating that acTS will not be a good source of macronutrients and trace elements. Microbial composition of bacteria, archaea, and fungi, and metabolomics analysis showed that acTS can provide some probiotics and some beneficial metabolites for human. It is therefore possible to develop acTS it into fermented food, functional food or oral liquid, only if when safeness of each microorganism is proved.

## CONFLICT OF INTEREST

All authors declare that they have no conflicts of interest.

6

**TABLE 4:** KEGG pathway and some related metabolites

KEGG pathway	Number	Metabolites
Nuclear receptors	4	Cortisone, Estradiol, Hydrocortisone, Estriol
Membrane transport	12	Octopine, Octopine, *D‐*Mannitol, Carnitine, *L‐*Phenylalanine, Choline, Inositol, Betaine, *L*‐Ascorbate, *D*‐Glucose‐6‐phosphate, Sucrose, Biotin
Signal transduction	12	Quercetin, Salicylic acid, Citric acid, *D‐*Glucose 6‐phosphate, *L‐*Ascorbate, acetoacetate, Serotonin, Phosphoethanolamine, Adenosine, Jasmonic acid, Acetylcholine, Inositol
Translation	5	*L*‐Phenylalanine, *L*‐Histidine, *L*‐Tyrosine, *L*‐Tryptophan, *L*‐Glutamic acid
Amino acid metabolism	51	Octopine, Carnitine, Kynurenic acid, 6‐Hydroxymelatonin, Serotonin, *L*‐Cystathionine, Urocanic Acid, Betaine, Creatinine, Phenylacetylglutamine, *L‐*Phenylalanine, Indole, Tyramine, Pipecolic acid, *L‐*Dopa, *L‐*Tryptophan, Sarcosine, *L‐*Glutamic acid, acetoacetate, 4‐Hydroxyphenylacetic acid, Kinic Acid, Choline, Hydroquinone, Octopine, Phenethylamine, Citric acid, *N*‐Acetyl*‐L‐*phenylalanine, 2‐Isopropylmalic acid, Protocatechuic acid, *L‐*Hydroxyproline, *N*‐Acetyl‐*D*‐phenylalanine, Capsaicin, Homoserine, Shikimic acid, Tyrosol, Homogentisic acid, Succinic acid, 2,5‐Dihydroxybenzaldehyde, Xanthurenic Acid, Phenol, *trans‐*Cinnamic acid, Phenylacetaldehyde, Salicylic acid, 3‐Methoxytyramine, *L‐*Tyrosine, Fumaric Acid, Rosmarinic Acid, Pipecolinic Acid, *L‐*Saccharopine, Phenylpyruvic Acid, Methylmalonic acid, 4‐Hydroxyphenylethanol
Biosynthesis of other secondary metabolites	44	*L‐*Tryptophan, Kaempferol, Scopolamine, Caffeine, 7‐Methylxanthine, Scopoletin, Coniferin, Theophylline, Pinocembrin, 3,4‐Dihydroxybenzaldehyde, Biochanin A, *L‐*Phenylalanine, Indole, Tyramine, Pipecolic acid, *L‐*Dopa, Catharanthine, Colchicine, Xanthine, Galangin, *L‐*Glutamic acid, Galangin, Papaverine, Inositol, Puromycin, Chrysin, Ecgonine, Piceatannol, Glycitein, Ferulic acid, Naringeninchalcone, Senecionine, Morphine, Quercetin, *L‐*Tyrosine, Luteolin, Phenylpyruvic Acid, *D‐*Glucose‐6‐phosphate, *trans‐*Cinnamic acid, Xanthohumol, Chlorogenic acid, Formononetin, Naringenin, Pipecolinic Acid, Genistein, 3‐Methoxytyramine
Chemical structure transformation maps	43	Octopine, Kaempferol, Catharanthine, Caffeine, Scopolamine, Serotonin, 3,4‐Dihydroxybenzaldehyde, Genistein, 3‐Methoxytyramine, Theophylline, Ferulic acid, Tyramine, Biochanin A, Scopoletin, *L‐*Phenylalanine, Colchicine, Pipecolic acid, Papaverine, Xanthine, *trans‐*Cinnamic acid,4‐Hydroxybenzaldehyde, *L‐*Glutamic acid, *L‐*Tryptophan, Jasmonic acid, Shikimic acid, *L‐*Dopa, IMP, Pipecolinic Acid, *L‐*Tyrosine, Naringeninchalcone, Senecionine, Morphine, Mevalonic acid, Phenylpyruvic Acid, Fumaric Acid, Succinic acid, Capsaicin, Formononetin, Naringenin, Protocatechuic acid, Citric acid, Gallic acid, 2,3‐Dihydroxybenzoic acid, Salicylic acid, *D‐*Glucose‐6‐phosphate,
Lipid metabolism	20	Estradiol, Thromboxane B2, Deoxycorticosterone, Jasmonic acid, Cortisone, Adrenosterone, Hydrocortisone, Prostaglandin B2, 2‐Methoxyestrone acetoacetate, Choline, Acetylcholine, Tetrahydrocorticosterone, Tetrahydrocortisone, Lignoceric acid, Citric acid, Arachidic acid, estrone 3‐sulfate, Eicosapentaenoic acid, Phosphoethanolamine
Metabolism of cofactors and vitamins	17	Vitamin A, 3‐Succinoylpyridine, Uracil, 4‐Pyridoxic acid, Desthiobiotin, *L‐*Glutamic acid, Pyridoxine, Biotin, Pantothenic acid, Dihydrouracil, Porphobilinogen, *L*‐Tyrosine, *trans*‐Cinnamic acid, Homogentisic acid, Succinic acid, Fumaric Acid, Pyridoxamine 5‐phosphate
Metabolism of terpenoids and polyketides	9	Adenine, Kanosamine, Eucalyptol, Carvone, Mevalonic acid, *L*‐Tyrosine, Gibberellic acid, Perillic acid, Salicylic acid
Digestive system	22	Vitamin A, Thromboxane B2, Octopine, Paracetamol, Pantothenic acid, *L‐*Phenylalanine, Choline, Serotonin, Hydrocortisone, Indole, Acetylcholine, Biotin, Tyramine, *L‐*Tryptophan, Phenol, 4‐Methylphenol, Sucrose, Salicylic acid, 4‐Methylphenol, Salicylic acid, Pyridoxamine, 5‐phosphate, D‐Glucose 6‐phosphate, estrone 3‐sulfate, *L*‐Ascorbate, Phenol, Sucrose, estrone 3‐sulfate, Pyridoxamine, 5‐phosphate, *D‐*Glucose 6‐phosphate, *L‐*Ascorbate

## Supporting information

Supporting Information
