# Supplementary material for: Chemical, microbial, and metabolic analysis of Taisui cultured in honey solution
Source: Food Sci Nutr. 2021 Feb 18;9(4):2158–68. doi: 10.1002/fsn3.2185 (PMC8020961; doi:10.1002/fsn3.2185)
Supplement: Supplementary file 1 — Supporting Information [file FSN3-9-2158-s001.docx]

**Supporting Information**

**Chemical, Microbial and Metabolomic Analysis of Taisui Cultured in Honey Solution**

Yunjing Chen, Shuxiu Zheng, Guangwen Zhang^*^, Jianming Luo, Junsheng Liu and Xichun Peng^*^

Department of Food Science and Engineering, Jinan University, Guangzhou, 510632, China.

^*^Correspondence

Guangwen Zhang and Xichun Peng, Department of Food Science and Engineering, Jinan University, Guangzhou, 510632, China. Emails: [tzgw@jnu.edu.cn](mailto:tzgw@jnu.edu.cn), [tpxchun@jnu.edu.cn](mailto:tpxchun@jnu.edu.cn)

Chen and Zheng contributed equally to this paper.

**Figure S1** Venn diagrams of bacteria (a), archaea (b) and fungi (c) in different groups. U: the upper part of Taisui; L: the lower part of Taisui; M: the cultured medium of Taisui.

**Figure S2** KEGG pathway annotation. Note: The abscissa represented the number of metabolites, and the ordinate represented the annotated KEGG pathway. The figure showed the number of metabolites annotated by each secondary classification under the pathway primary classification.

**Table S1** Relative abundance (RA) of bacteria, archaea and fungi at phylum level in different groups.

**Table S2** Relative abundance (RA) of bacteria, archaea and fungi at genus level of different groups.

**Table S3** Information of some metabolites


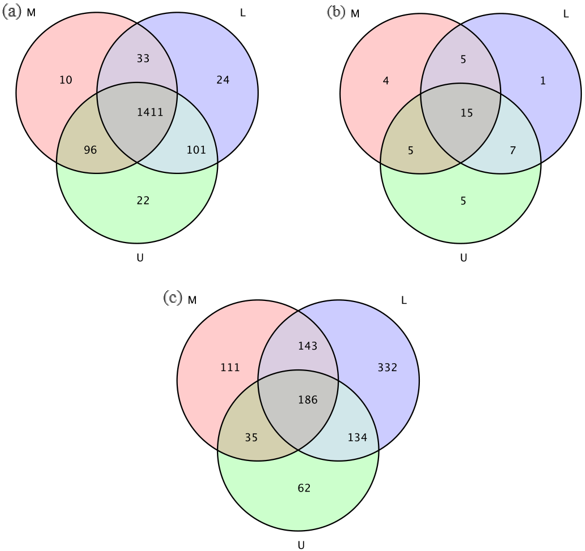


**Figure S1** Venn diagrams of bacteria (a), archaea (b) and fungi (c) in different groups. U: the upper part of Taisui; L: the lower part of Taisui; M: the cultured medium of Taisui.


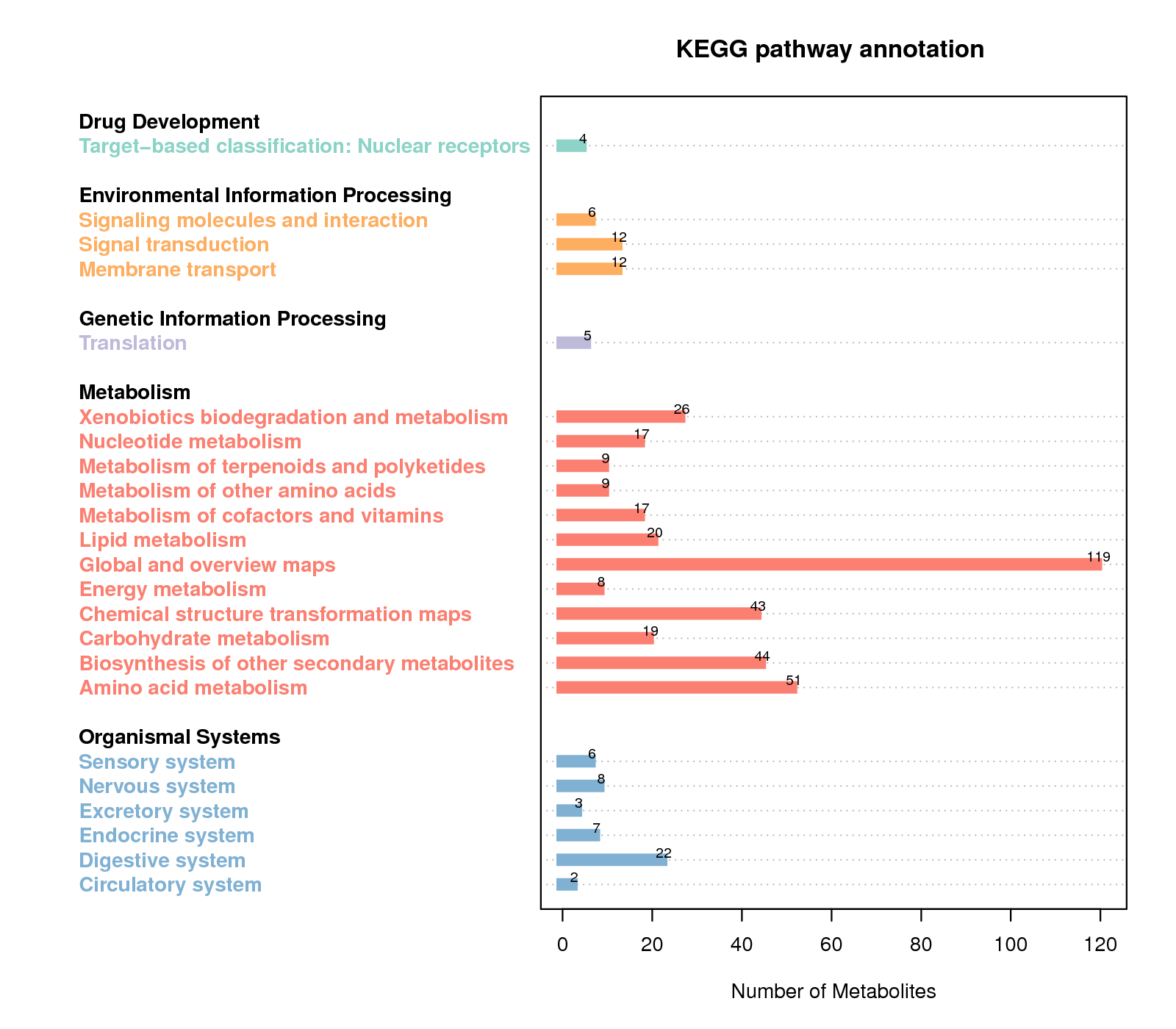


**Figure S2** KEGG pathway annotation. Note: The abscissa represented the number of metabolites, and the ordinate represented the annotated KEGG pathway. The figure showed the number of metabolites annotated by each secondary classification under the pathway primary classification.

**Table S1** Relative abundance (RA) of bacteria, archaea and fungi at phylum level in different groups

| U | RA/% | L | RA/% | M | RA/% |
| --- | --- | --- | --- | --- | --- |
| Bacteria | | | | | |
| Proteobacteria | 43.01±1.13 | Proteobacteria | 38.73±2.34 | Proteobacteria | 41.70±0.31 |
| Chloroflexi | 13.38±0.85 | Firmicutes | 15.29±1.36 | Chloroflexi | 13.16±0.50 |
| Firmicutes | 11.11±0.24 | Chloroflexi | 12.20±0.15 | Firmicutes | 10.65±0.76 |
| Acidobacteria | 7.55±0.55 | Acidobacteria | 7.20±0.20 | Acidobacteria | 8.44±0.48 |
| Actinobacteria | 7.34±0.19 | Actinobacteria | 7.17±0.18 | Actinobacteria | 8.29±1.38 |
| Bacteroidetes | 5.40±0.49 | Bacteroidetes | 7.15±1.02 | Bacteroidetes | 5.00±0.06 |
| Nitrospirae | 4.30±0.13 | Nitrospirae | 4.27±0.22 | Nitrospirae | 4.77±0.25 |
| Gemmatimonadetes | 1.07±0.10 | Verrucomicrobia | 1.58±0.50 | Nitrospinae | 0.97±0.25 |
| Nitrospinae | 0.97±0.05 | Gemmatimonadetes | 0.93±0.07 | Gemmatimonadetes | 0.90±0.05 |
| Cyanobacteria | 0.84±0.15 | Planctomycetes | 0.85±0.01 | Cyanobacteria | 0.84±0.12 |

Note: U: the upper part of Taisui; L: the lower part of Taisui; M: the cultured medium of Taisui.

**Table S1** Relative abundance (RA) of bacteria, archaea and fungi at phylum level in different groups (Continued)

| U | RA/% | L | RA/% | M | RA/% |
| --- | --- | --- | --- | --- | --- |
| Archaea | | | | | |
| Euryarchaeota | 93.34±4.68 | Euryarchaeota | 88.61±0.72 | Euryarchaeota | 98.07±0.87 |
| Crenarchaeota | 6.42±4.59 | Crenarchaeota | 9.01±4.00 | Crenarchaeota | 1.04±1.47 |
|  |  | Diapherotrites | 2.38±2.37 |  |  |
| Fungi | | | | | |
| Ascomycota | 72.91±30.50 | Ascomycota | 46.56±3.34 | Ascomycota | 87.24±1.74 |
| Basidiomycota | 1.77±1.21 | Rozellomycota | 19.09±1.45 | Basidiomycota | 6.05±1.66 |
| Mortierellomycota | 0.50±0.39 | Basidiomycota | 7.47±0.31 | Mortierellomycota | 2.09±0.08 |
| Rozellomycota | 0.05±0.02 | Mortierellomycota | 3.45±0.19 | Zoopagomycota | 0.20±0.09 |
| Chytridiomycota | 0.02±0.01 | Chytridiomycota | 0.25±0.04 | Rozellomycota | 0.15±0.10 |
| Zoopagomycota | 0.02±0.01 | Zoopagomycota | 0.15±0.05 | Chytridiomycota | 0.14±0.09 |

Note: U: the upper part of Taisui; L: the lower part of Taisui; M: the cultured medium of Taisui.

**Table S2** Relative abundance (RA) of bacteria, archaea and fungi at genus level of different groups

| U | RA/% | L | RA/% | M | RA/% |
| --- | --- | --- | --- | --- | --- |
| Bacteria | | | | | |
| *uncultured_bacterium_f_Anaerolineaceae* | 6.72±0.35 | *uncultured_bacterium_f_Anaerolineaceae* | 6.15±0.04 | *uncultured_bacterium_f_Anaerolineaceae* | 6.77±0.77 |
| *Alcaligenes* | 4.94±0.81 | *Alcaligenes* | 4.40±0.06 | *Alcaligenes* | 5.48±0.28 |
| *Ochrobactrum* | 3.90±0.22 | *Lactobacillus* | 4.25±0.94 | *Ochrobactrum* | 3.92±0.11 |
| *uncultured_bacterium_c_Thermodesulfovibrionia* | 3.67±0.11 | *Ochrobactrum* | 4.00±0.12 | *uncultured_bacterium_c_Thermodesulfovibrionia* | 3.91±0.25 |
| *uncultured_bacterium_c_Subgroup_6* | 3.27±0.23 | *uncultured_bacterium_c_Thermodesulfovibrionia* | 3.55±0.09 | *uncultured_bacterium_c_Subgroup_6* | 3.38±0.27 |
| *uncultured_bacterium_f_Steroidobacteraceae* | 3.04±0.13 | *uncultured_bacterium_c_Subgroup_6* | 3.10±0.14 | *Methylotenera* | 2.92±0.34 |
| *Methylotenera* | 2.97±0.09 | *uncultured_bacterium_f_Steroidobacteraceae* | 2.69±0.21 | *uncultured_bacterium_f_Steroidobacteraceae* | 2.83±0.53 |
| *Lactobacillus* | 2.61±0.28 | *Methylotenera* | 2.69±0.02 | *Lactobacillus* | 2.29±0.19 |
| *Ralstonia* | 1.98±0.28 | *uncultured_bacterium_f_Muribaculaceae* | 2.55±0.90 | *uncultured_bacterium_f_SC-I-84* | 1.89±0.20 |
| *uncultured_bacterium_f_SC-I-84* | 1.76±0.08 | *Ralstonia* | 1.65±0.77 | *Ralstonia* | 1.58±0.15 |

Note: U: the upper part of Taisui; L: the lower part of Taisui; M: the cultured medium of Taisui.

**Table S2** Relative abundance (RA) of bacteria, archaea and fungi at genus level of different groups (Continued)

| U | RA/% | L | RA/% | M | RA/% |
| --- | --- | --- | --- | --- | --- |
| Archaea | | | | | |
| *Methanosaeta* | 32.03±2.53 | *Methanosaeta* | 30.11±8.47 | *Methanosaeta* | 35.35±21.60 |
| *Methanosphaera* | 20.63±1.44 | *Natronomonas* | 23.75±6.91 | *Natronomonas* | 20.40±17.30 |
| *Natronomonas* | 17.59±5.07 | *Methanolinea* | 10.15±4.76 | *Methanobrevibacter* | 10.97±12.05 |
| *uncultured_bacterium_c_Bathyarchaeia* | 6.42±4.59 | *uncultured_bacterium_c_*  *Bathyarchaeia* | 9.01±4.00 | *Methanobacterium* | 7.03±8.43 |
| *Methanobacterium* | 5.89±3.99 | *uncultured_bacterium_f_*  *Methanomassiliicoccaceae* | 7.85±5.58 | *Methanosphaera* | 6.92±9.78 |
| *Methanobrevibacter* | 4.91±6.91 | *Methanobrevibacter* | 4.28±3.11 | *Candidatus_*  *Methanoplasma* | 3.92±5.06 |
| *Methanosarcina* | 3.60±5.09 | *Methanosarcina* | 3.90±5.52 | *Methanosarcina* | 3.35±4.74 |
| *Methanospirillum* | 3.04±3.33 | *Methanosphaera* | 3.59±5.08 | *Methanolinea* | 2.73±3.69 |
| *Methanolinea* | 1.99±2.75 | *Methanomassiliicoccus* | 2.71±3.38 | *Halopenitus* | 2.14±1.89 |
| *Haloparvum* | 1.79±1.57 | *uncultured_bacterium_c_Micrarchaeia* | 2.38±3.37 | *Methanomassiliicoccus* | 1.95±1.46 |

Note: U: the upper part of Taisui; L: the lower part of Taisui; M: the cultured medium of Taisui.

**Table S2** Relative abundance (RA) of bacteria, archaea and fungi at genus level of different groups (Continued)

| U | RA/% | L | RA/% | M | RA/% |
| --- | --- | --- | --- | --- | --- |
| Fungi | | | | | |
| *Zygosaccharomyces* | 63.17±27.71 | *Zygosaccharomyces* | 8.23±4.17 | *Zygosaccharomyces* | 66.61±3.64 |
| *Plectosphaerella* | 0.72±0.40 | *Dekkera* | 3.42±0.66 | *Mortierella* | 1.86±0.15 |
| *Fusarium* | 0.57±0.52 | *Mortierella* | 3.33±0.15 | *Plectosphaerella* | 1.59±0.33 |
| *Cladosporium* | 0.53±0.32 | *Fusarium* | 2.31±0.11 | *Malassezia* | 1.37±0.30 |
| *Mortierella* | 0.44±0.33 | *Cladosporium* | 1.47±0.03 | *Fusarium* | 1.28±0.30 |
| *Malassezia* | 0.41±0.30 | *Penicillium* | 1.45±0.12 | *Cladosporium* | 1.21±0.05 |
| *Candida* | 0.40±0.29 | *Debaryomyces* | 1.42±0.00 | *Aspergillus* | 0.92±0.25 |
| *Penicillium* | 0.28±0.08 | *Candida* | 1.31±0.17 | *Lactarius* | 0.60±0.64 |
| *Chaetomium* | 0.21±0.23 | *Alternaria* | 1.07±0.10 | *Candida* | 0.56±0.22 |
| *Pseudopithomyces* | 0.20±0.23 | *Pichia* | 1.04±0.13 | *Pseudopithomyces* | 0.53±0.05 |

Note: U: the upper part of Taisui; L: the lower part of Taisui; M: the cultured medium of Taisui.

**Table S3** Information of some metabolites

| Metabolites | FC (Cul/Con) | Metabolites | FC (Cul/Con) | Metabolites | FC (Cul/Con) |
| --- | --- | --- | --- | --- | --- |
| Citric acid | 256.01 | Kinic Acid | 26.26 | Guanine | 8.31 |
| Phenethylamine | 114.4 | Cytosine | 23.04 | Chlorogenic acid | 7.87 |
| *L*- henylalanine | 90.76 | Hypoxanthine | 22.86 | Gallic acid | 6.91 |
| *D*-Glucono-1,5-lactone | 86.47 | 3-Methoxytyramine | 17.34 | 4-Pyridoxic acid | 6.74 |
| Styrene | 75.31 | Uracil | 15.6 | 4-Hydroxybenzaldehyde | 6.63 |
| Adenine | 63.48 | Pyridoxamine 5-phosphate | 13.83 | 4-Methylphenol | 6.26 |
| *trans*-Aconitic acid | 47.9 | Piceatannol | 10.7 | Puromycin | 5.43 |
| Desthiobiotin | 46.22 | Adrenosterone | 10.37 | Protocatechuic acid | 5.39 |
| Thymine | 33.48 | Pantothenic acid | 9.8 | 2-Furoic acid | 4.74 |
| Salicylic acid | 31.71 | Cytidine | 9.39 | Cuminaldehyde | 4.62 |
| *L*-Saccharopine | 31.17 | 2-Methoxyestrone | 9.26 | *L*-Homoserine | 4.56 |
| *L*-Glutamic acid | 27.81 | Succinic acid | 8.6 | Quercetin | 4.53 |

**Table S3** Information of some metabolites (Continued)

| Metabolites | FC (Cul/Con) | Metabolites | FC (Cul/Con) | Metabolites | FC (Cul/Con) |
| --- | --- | --- | --- | --- | --- |
| Jasmonic acid | 3.99 | Coniferin | 2.63 | Phenylacetaldehyde | 0.2 |
| 4-Hydroxyphenylethanol | 3.94 | Sucrose | 2.62 | *trans*-Cinnamic acid | 0.19 |
| Acetylcholine | 3.59 | Thromboxane B2 | 2.62 | *D*-Mannitol | 0.14 |
| Kynurenic acid | 3.51 | *o*-Cresol | 2.61 | Tetrahydrocortisone | 0.14 |
| Ferulic acid | 3.44 | 4-Hydroxyphenylacetic acid | 2.59 | Senecionine | 0.13 |
| Sarcosine | 3.34 | Fumaric Acid | 2.48 | Stachyose | 0.12 |
| Itaconic acid | 3.24 | 2,3-Dihydroxybenzoic acid | 2.26 | Papaverine | 0.11 |
| Terephthalic acid | 3.22 | Genistein | 2.26 | Catharanthine | 0.1 |
| Hydrocortisone | 3.02 | 3,4- Dihydroxybenzaldehyde | 2.25 | Phenol | 0.1 |
| Porphobilinogen | 2.95 | Luteolin | 2.23 | *L*-Cystathionine | 0.08 |
| Vanillyl alcohol | 2.83 | Naringenin | 2.22 | Tyramine | 0.06 |
| *L*-Ascorbate | 2.66 | Kaempferol | 2.2 | Scopolamine | 0.05 |
